# Moderate Iodine Deficiency Is Common in Pregnancy but Does Not Alter Maternal and Neonatal Thyroid Function Tests

**DOI:** 10.3389/fendo.2020.523319

**Published:** 2020-12-11

**Authors:** Tal Schiller, Arnon Agmon, Viviana Ostrovsky, Gabi Shefer, Hilla Knobler, Taiba Zornitzki

**Affiliations:** ^1^Diabetes, Endocrinology and Metabolic Disease Institute, Kaplan Medical Center, Hebrew University Medical School, Rehovot, Israel; ^2^Clalit Health Services, Lev Tel Aviv Women’s Health Center, Tel Aviv, Israel; ^3^Tel Aviv Sourasky Medical Center, Institute of Endocrinology, Metabolism and Hypertension, Tel Aviv, Israel

**Keywords:** urine iodine content, thyroid, pregnancy, neonate, thyroid function

## Abstract

**Introduction:**

An Israeli national survey found that 85% of pregnant women had urinary iodine content (UIC) levels below the adequacy range (<150 µg/L). Widespread desalinated water usage and no national fortification plan are possible causes. Studies assessing relationships between iodine status and maternal and neonatal thyroid function provided varying results. Our aims were to determine whether iodine deficiency was associated with altered maternal or neonatal thyroid function and the factors leading to iodine deficiency.

**Methods:**

A cross-sectional study including 100 healthy women without prior thyroid disease, in their first trimester of a singleton pregnancy were recruited from an HMO clinic in central Israel. The women were followed from their first trimester. All women completed a 24-h dietary recall and life habits questionnaires. We tested for UIC, maternal and neonatal thyroid function, maternal autoantibodies, and neonatal outcomes.

**Results:**

Median UIC in our cohort was 49 µg/L [25%–75% interquartile range (IQR) 16-91.5 µg/L], with 84% below adequacy range. No correlation was found between iodine deficiency and maternal or neonatal thyroid function which remained within normal ranges. Antibody status did not differ, but thyroglobulin levels were significantly higher in iodine insufficient subjects. UIC was higher in women consuming an iodine containing supplement. There was no association between UIC and dietary iodine content or water source.

**Conclusions:**

Moderate iodine deficiency is common in our healthy pregnant women population. Our data imply that moderate iodine deficiency in pregnancy seem sufficient to maintain normal maternal and neonatal thyroid function.

## Introduction

Iodine deficiency is a global problem and is considered to have substantial implications during pregnancy (1). During pregnancy, iodine requirements increase significantly to enable synthesis of sufficient amounts of thyroid hormones to meet fetal and maternal needs (2). The US Institute of Medicine (IOM) and the World Health Organization (WHO) recommend for pregnant women a daily iodine consumption of 220 and 250 mcg, respectively. The recommended method to determine iodine consumption in a population is by urinary iodine concentration (UIC) in spot urine specimens and levels of 150–249 µg/L are considered adequate (1). According to the American Thyroid Association (ATA) guidelines from 2017 pregnant women whose median UICs are 50 to 150 µg/L are defined as mildly to moderately iodine deficient (2).

The first national survey determining iodine status in the Israeli population was conducted in 2017 and examined iodine status among school-aged children and pregnant women (3). The results showed mild to moderate iodine deficiency in both groups and 85% of pregnant women had UIC levels below the adequacy range with a median of 61 µg/L (IQR 36–97 µg/L). Heavy reliance on desalinated water and the lack of a national fortification plan were suggested as possible causes.

The grave consequences of severe iodine deficiency are well established and include maternal and fetal hypothyroidism and impaired neurological fetal development (1). Severely iodine deficient populations show clear benefits from iodine supplementation (1). However, the short- and long-term implications of mild to moderate iodine deficiency are still debated (4).

Several studies conducted in recent years provide conflicting data regarding the correlation between urinary iodine levels and maternal thyroid function parameters (5–8) and there is limited data on the relationship between neonatal thyroid function tests and the mother’s iodine status. Previous studies did not reveal a significant association (7, 9). However, possible long-term neurodevelopmental adverse effects of mild to moderate iodine deficiency have been suggested by other observational studies (10–12). Although the recent 2017 guidelines from ATA still recommend iodine supplementation to pregnant and lactating women, there is uncertainty regarding the benefit of iodine supplements in mild to moderate iodine deficient women (8, 13).

The aims of the present study are: 1) To evaluate the iodine status determined by UIC in a cohort of healthy pregnant women who have no previous history of thyroid disease; 2) To determine whether iodine deficiency is associated with altered thyroid functions in mothers or their newborns or with maternal thyroid autoimmunity; 3) To determine the factors leading to iodine deficiency including dietary iodine content, main water source and consumption of iodine containing supplements; 4) To assess the relationship between UIC and birth outcomes.

## Methods

This cross-sectional study included 100 healthy women in their first trimester of a singleton pregnancy who were recruited from a large HMO clinic in central Israel. The women were followed from their first trimester through birth. The study was approved by the Institutional Review Board (IRB) of Clalit Health Services, the largest Israeli HMO organization and all participants signed an informed consent form.

Exclusion criteria were: 1) Known previous thyroid disease; 2) Any significant background illness; 3) Exposure to iodine containing contrast agents in the previous year before study enrollment; 4) Exposure to any iodine containing medications except multivitamin and mineral supplements.

Out of 116 consecutive women screened, 10 declined participation and the other six were excluded because of a known thyroid disease or other significant background illness. Thus, 100 women were included in the final analysis.

All women filled detailed questionnaires including the following: demographic data, anthropometric data, personal relevant medical history and thyroid illnesses in their first-degree relatives, current smoking status, medications including iodine containing supplements consumption and a 24-h dietary recall questionnaire that included major iodine containing food groups including iodinated salt, dairy products, eggs, bread, fish, shellfish, and seaweed. Soy products were also included due to their goitrogenic effect. We also asked about the main water source (mineral/tap water) prior to the study entry. Six women were excluded from 24-h recall analysis due to insufficient data. The questionnaire was adapted from Leung et al. (14), translated to Hebrew with a few changes made to adjust for local eating habits.

Total iodine intake was calculated excluding iodine containing supplements and divided according to WHO adequacy range to below 250 µg and above 250 µg daily considered adequate.

### Maternal Thyroid Function Tests

Blood tests for thyroid function and autoantibodies included: TSH, FT4, FT3, anti-thyroid peroxidase (TPO), anti-thyroglobulin (anti-Tg), and thyroglobulin (Tg). All samples were analyzed at a single certified laboratory. TSH (normal range 0.27–4.2 mU/L), FT4 (normal range 11–22 pmol/L), and FT3 (normal range 3.5–6.5 pmol/L) levels were analyzed by Centaur-XP (Siemens, Los Angeles, CA). Anti TPO (normal range <35 IU/L) by Immulite 1000 (Siemens, Los Angeles, CA) and Tg (normal range 1.2–50 ng/ml) and anti-Tg (normal range <4 IU/L) by Access (Beckman Culter, CA, USA).

### Urinary Iodine Content

Forty-five ml of urine were collected for UIC analysis. All tubes were covered in aluminum foil to protect from light exposure as required and frozen within half an hour at -20^0^C. The tubes were then transferred to a certified central lab for analysis. Analysis was done by cleaning the urine with a solid phase ion exchange and subsequently iodine was measured by a Thermo ISE (ion selective electrode) (Thermo Fisher Scientific, MA, USA), with a lower limit of detection of 6 µg (15). Fourteen randomly selected urine samples were also analyzed by HPLC (High Performance Liquid Chromatography) with an excellent correlation (r=0.96) (16, 17).

### Neonatal Thyroid Function Tests

Data from the national neonatal Total Thyroxine (TT4) (µg/dl) screening was used for assessing newborns’ thyroid status. All tests were done at a single central laboratory in Israel. All newborns were screened for TT4 levels on their 2^nd^ day of life. According to a national policy, in specimens demonstrating TT4 levels at the lowest daily 10^th^ percentile, TSH levels were also determined.

### Statistical Analysis

Data are presented as median or percentages. Continuous variables were tested for normality using a Shapiro-Wilk test and when an abnormal distribution was found, non-parametric tests were performed. The Mann-Whitney test was performed to compare two groups and the Kruskal-Wallis test was used to compare three groups or more. Categorical variables were compared using the Pearson’s chi-squared test. Receiver operating characteristic (ROC) curve analysis was performed to determine the best discriminatory Tg level cutoff for iodine deficiency identification. P values <0.05 were considered statistically significant. Data were analyzed using SPSS21.

The sample size was designed to detect a 10% difference in FT4 levels between UIC < 50 µg/L and UIC >150 µg/L at a significance of 5% with a power of 80%.

## Results

### Study Population

Women included in the study were recruited in the first trimester at a mean gestational age of 11 + 4 (SD ± 1.7) weeks of gestation. The median UIC in our cohort was 49 µg/L (IQR 16-91 µg/L) and 84% of the women had UIC levels below adequacy range. Baseline characteristics of women according to UIC are summarized in **Table 1**. None of the variables accounted for the difference in UIC except for reported consumption of iodine containing supplements: 94% of women with adequate UIC used an iodine containing supplement (the most prevalent supplement in Israel contains 220 mcg iodine per tablet) compared with about half of the women with iodine deficiency. As shown, most women drank mineral water as their main water source. Calculated median iodine intake in the entire cohort was 186mcg iodine daily which is considered insufficient, but we could not demonstrate a difference in UIC levels between those who consumed more than 250 mcg daily to those who consumed less than 250 mcg daily.

**Table 1:** Baseline characteristic of the study population stratified by UIC categories.

Variable	Total	<100 mcg/L (N = 77)	100–150 mcg/L (N = 7)	>150 mcg/L (N = 16)	P value
Age, years (mean) ± SD	32 ± 5	32.4 ± 5.6	31.7 ± 3.7	30.7 ± 4.1	0.508
Gestational age at sampling, weeks (mean) ± SD	11.4 ± 1.7	11.3 ± 1.8	11.4 ± 1.7	11.7 ± 1.7	0.864
High school education, N	43	33	3	7	0.965
Born in Israel, N	83	67	4	12	**0.049**
BMI, kg/m^2^ (mean) ± SD	23 ± 4.4	22.8 ± 4.2	26.9 ± 8.4	22.7 ± 2.7	0.332
Nulliparous, N	37	30	2	5	0.691
Smoker, N	12	9	2	1	0.312
Positive family history of thyroid disease, N	14	10	0	4	0.248
Iodine containing supplements consumption, N	60	41	3	16	**0.008**
24-h recall ≤ 250 µg/day, N*	76	59	4	13	0.575
24-h recall > 250 µg/day, N*	18	14	2	2	
Tap water as main drinking source, N	14	10	0	4	0.289

*94 questionnaires included.

UIC, urinary iodine concentration; SD, standard deviation; BMI, body mass index.

Bolded values are those that were statistically significant.

### Maternal and Neonatal Thyroid Function Tests

**Figure 1:**
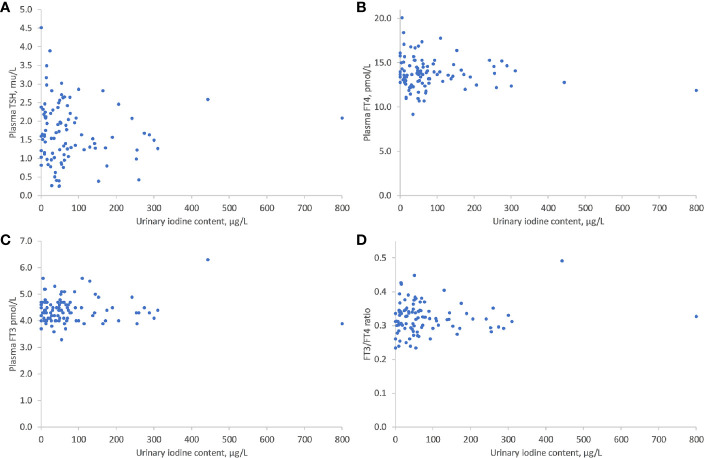
Correlations between UIC and TSH **(A)**, FT4 **(B)**, FT3 **(C)**, and T3/T4 **(D)**. UIC, Urinary iodine concentration; TSH, thyroid stimulating hormone; FT4, free thyroxine; FT3, free triiodothyronine.

**Figure 2:**
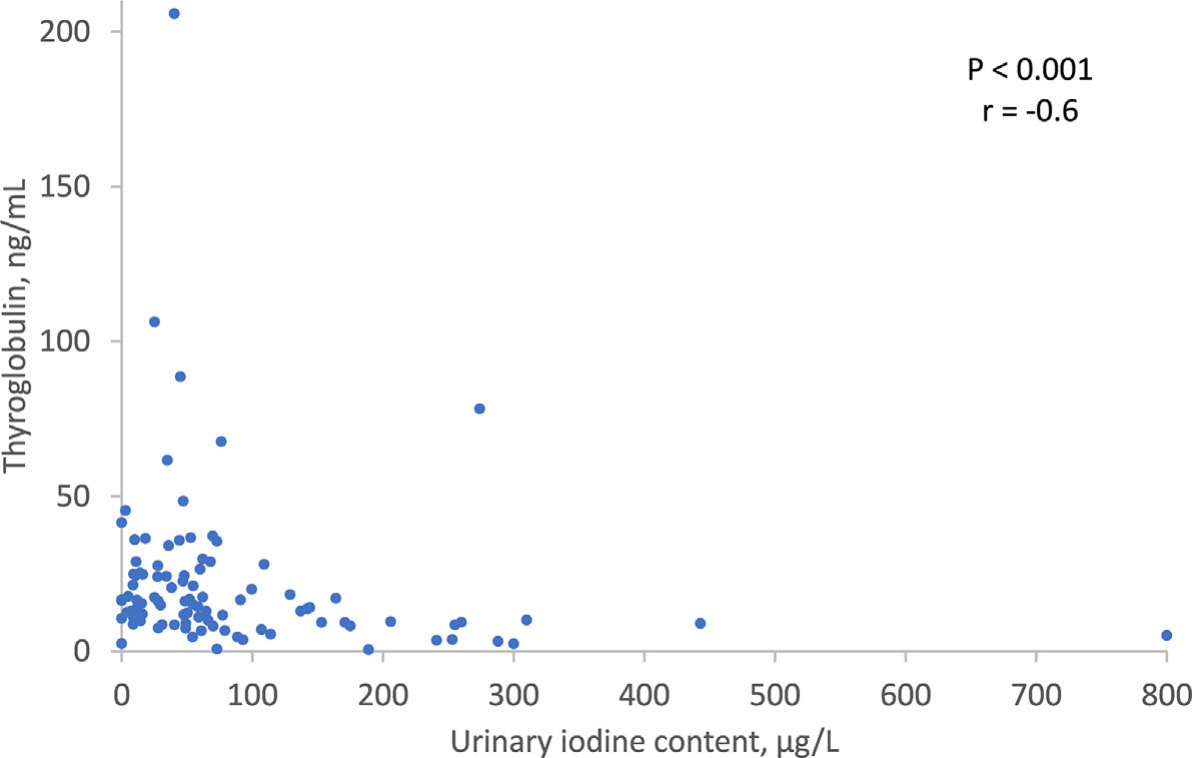
Correlation between UIC and Thyroglobulin. UIC, Urinary iodine concentration.

**Figure 3:**
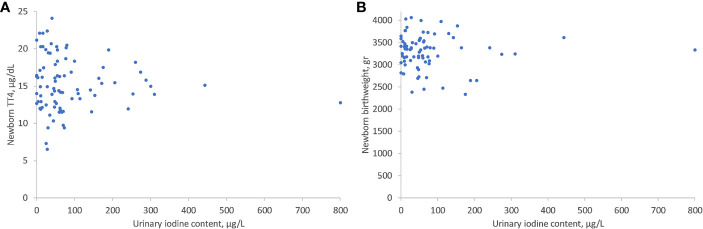
Correlations between UIC and neonatal TT4 **(A)** and neonatal birth weight **(B)**. UIC, Urinary iodine concentration; TT4, Total T4.

As shown in **Figure 1**, there was no significant correlation between UIC levels and maternal thyroid function tests: TSH (r = -0.277, P = 0.449), FT4 (r= -0.207, P=0.675), FT3 (r= -0.114, P=0.899), or FT3/FT4 ratio (r= 0.104, P= 0.303) (**Figure 1**). There was, however, a significant inverse correlation between lower UIC and higher Tg levels (r = -0.6, P < 0.001) (**Figure 2**). Based on ROC curve analysis, a Tg cut off level of above 10.1 ng/ml was associated with 79% risk of being iodine deficient (<100 µg/L). Similarly, no correlation was found between UIC and neonatal TT4 (r= -0.217, P =0.667) (**Figure 3**).

When divided into UIC categories as shown in **Table 2**, no association was demonstrated between the three UIC categories and either maternal or neonatal thyroid function tests. Furthermore, thyroid function tests remained well within the normal range even in the lowest UIC category. Thirteen percent of our cohort was thyroid antibody positive. However, no difference was found in antibody positivity between the UIC groups.

**Table 2:** Maternal and neonatal thyroid function tests stratified by UIC categories.

Variable	Total (N=100)	<100 mcg/L (N=77)	100–150 mcg/L (N=7)	>150 mcg/L (N=16)	P value
TSH, mU/L	1.6 ± 0.82	1.7 ± 0.9	1.4 ± 0.2	1.5 ± 0.7	0.511
FT4, pmol/L	13.7 ± 1.8	13.7 ± 1.8	13.6 ± 1.7	13.8 ± 1.3	0.851
FT3, pmol/L	4.4 ± 0.5	4.4 ± 0.4	4.5 ± 0.7	4.3 ± 0.6	0.452
*Antibody positive, N	13	10	1	2	0.993
Thyroglobulin, ng/ml	14.9 ± 25.8	16.6 ± 27.7	13.7 ± 7.5	8.8 ± 18.2	**<0.001**
Neonatal TT4, µg/dl	14.9 ± 3.6	14.9 ± 3.9	14 ± 1.2	15.4 ± 2.1	0.492

*Antibody positive includes women that were either anti-TPO positive and/or anti-thyroglobulin positive.

Data presented as median ± SD.

TSH, thyroid stimulating hormone; FT4, free T4; FT3, free T3; TPO, thyroid peroxidase; SD, standard deviation.

Bolded values are those that were statistically significant.

One woman in the entire cohort was found to have newly diagnosed hypothyroidism due to Hashimoto’s disease and treatment was initiated. One other woman had TSH levels less than 0.1mU/L that resolved during follow up.

### Thyroid Function According to Iodine Containing Supplements Status

**Table 3** compares UIC, thyroid function tests, thyroid antibodies and Tg according to reported regular intake of iodine containing supplements versus those who did not. Even though iodine containing supplements consumption led to significantly higher UIC, it had no effect on maternal TSH, FT4, FT3, or neonatal TT4. However, there was a non-statistically significant trend of being thyroid-antibody positive in women who did not consume an iodine containing supplement and this group had higher Tg levels.

**Table 3:** Thyroid function tests, thyroid antibodies, and UIC stratified by iodine containing supplements status.

Variable	Yes (N=60)	No (N=40)	P value
UIC, µg/L	59 ± 134.6	39 ± 49.5	**0.043**
TSH, mU/L	1.6 ± 0.85	1.5 ± 0.8	0.427
FT4, pmol/L	13.7 ± 1.7	13.6 ± 1.8	0.6
FT3, pmol/L	4.3 ± 0.5	4.4 ± 0.4	0.78
Antibody Positive,N*	5	8	0.096
Thyroglobulin, ng/ml	12.6 ± 16.8	18.3 ± 34.8	**0.01**
Neonatal TT4, µg/dl	15 ± 3.3	14.5 ± 4.1	0.798

*Antibody positive includes women that were either anti-TPO positive and/or anti-thyroglobulin positive.

Data presented as median ± SD.

UIC, urinary iodine concentration; TSH, thyroid stimulating hormone; FT4, free T4; FT3, free T3; TPO, thyroid peroxidase; SD, standard deviation.

Bolded values are those that were statistically significant.

### Birth Outcomes

There was no correlation between UIC and week of birth (r= -0.228, P=0.653) or birth weight (r= -0.277, P=0.486) (**Figure 3**). The median week of delivery was 39 weeks (IQR 38–40) and the median birth weight was 3.37 Kg (IQR 3.09–3.56). When divided into UIC categories, we did not detect differences in any of these parameters between the lowest to the highest UIC groups.

Four abortions occurred during follow-up, three in the lowest UIC group and one in a woman with a UIC measurement of 255 µg/L. All of them occurred in the second trimester between the 15^th^ to the 22^nd^ week of pregnancy. In three of them, the pregnancy was terminated due to severe malformations (situs inversus, neural tube defect and hydrocephalus).

## Discussion

Our study evaluated a cohort of one hundred healthy, first trimester pregnant women. We found that 84% of the cohort had UIC levels below the WHO’s adequacy range for pregnancy (150–249 µg/L). These findings are in line with previous data derived from a larger survey evaluating iodine status in 1,074 pregnant Israeli women (3).

Despite the high prevalence of iodine deficiency in pregnant women, we did not find a correlation between iodine deficiency and maternal thyroid function tests: TSH, FT4, FT3, or FT3/FT4. There was also no correlation between iodine deficiency and the presence of autoantibodies. Previous studies evaluating the effect of moderate iodine deficiency on maternal and neonatal thyroid function provided inconsistent results. This inconsistency is related partly to variations in the studies’ population such as: exclusion of previous thyroid disease, timing of sampling during pregnancy, iodine status in each country reflecting on maternal iodine reserve, nutritional iodine consumption and compliance with iodine supplementation. However, most recent studies found only a modest effect or no effect of moderate iodine deficiency on maternal thyroid function. A national survey of pregnant women in Belgium found a high prevalence of thyroid disorders affecting one in six pregnant women and high prevalence of iodine deficiency (the median UIC was 117 µg/L (70–189 ug/L) in the first trimester and 131 (74–239) ug/L in the third trimester). However, there was no significant association between UIC and TSH and there was only a weak correlation with FT3 and FT4. Interestingly in iodine deficient women TSH levels (in parallel with Tg) increased more in the third trimester compared with iodine-sufficient women suggesting that iodine-deficient status leads to thyroidal hyper stimulation (6). In a study of healthy pregnant women in Spain in which about two thirds underwent evaluation in the first trimester and a third in the second trimester, they found that most women had iodine deficiency, but there was no relationship between UIC and maternal or neonatal thyroid function tests (7). An intriguing study from Norway showed that low UIC (<100 µg/L) was associated with elevated FT3 levels but not with elevated FT4 levels which may reflect preferential T3 secretion, a known auto- regulatory mechanism in iodine deficiency (8, 18). A recent study from Sri Lanka included 425 women evaluated in the first trimester and followed through the third trimester (5). Although in the first trimester UIC was adequate it decreased significantly in the third trimester to insufficiency (from 170 to 105 µg/L) and TSH increased from 1.3 to 1.6 µIU/ml. Albeit the increase was significant, TSH levels remained within the reference range (0.3–5.2 µIU/ml) and the implication of this change is questionable. Taken together, our data and most of previous studies’ data imply that moderate iodine deficiency in pregnancy seem sufficient to maintain maternal thyroid function within the normal range.

Similar to previous studies, we demonstrated a significant correlation between iodine deficiency and a higher Tg level probably reflecting an enlarged thyroid mass (5, 19). Based on a ROC curve analysis, a cut-off level of above 10.1 ng/ml was associated with a 79% risk of having iodine deficiency (<100 µg/L). Since UIC testing is an expensive test not readily available, Tg levels can serve as a surrogate marker for iodine deficiency in pregnancy.

Notably, maternal iodine deficiency did not affect neonatal thyroid function, birth week, or birth weight and was not associated with other significant adverse birth outcomes. Our results are in accordance with previous studies that did not find an association between UIC and neonatal TSH (7, 19). Similarly, several previous studies have found no association between UIC and birth outcomes, such as birth weight or preterm birth in iodine deficient women (19, 20). A study from Thailand did report significantly higher rates of preterm birth and low birth weight in the iodine insufficient group (21). In the later study women who took iodine supplements were excluded from the analysis. It is possible that iodine deficiency was accompanied by other nutritional deficits which could confound pregnancy outcomes.

Although our data and data derived from other studies regarding the short-term effects of mild-moderate maternal iodine deficiency on neonates is reassuring, the long-term adverse effects of iodine deficiency must be considered. Several observational cohort studies (9–11, 22, 23) demonstrated an association between iodine deficiency and neurodevelopmental delays. One of these studies showed that even mild iodine deficiency during pregnancy had a long-term adverse impact on fetal neuro-cognition, not ameliorated by iodine sufficiency during childhood (12). An additional study, also found that children born to mothers with lower UIC had lower IQ and reading accuracy (11). Mild iodine deficiency was associated with language delay, lower fine motor and communication skills, behavior problems and attention deficit disorders later in life (10, 22). Although the observational design of these studies limits definite conclusions, they raise some concern.

Iodine deficiency in Israel can be explained by the widespread use of desalinated water for home use, lack of a national fortification plan and low dairy product consumption (24). However, there was no association between UIC levels and iodine consumption, or with mineral water or iodized salt consumption. In addition, we did not find an association to smoking which might exacerbate iodine deficiency due to high thiocyanate levels. The only source that was significantly associated with adequate iodine levels was iodine containing supplements consumption which was reported by approximately two thirds of the women.

Iodine containing supplements usage, however, did not affect maternal or neonatal thyroid function tests except higher Tg levels in the group not taking supplements, reflecting lower UIC.

The need for iodine supplementation in mild to moderate iodine deficiency is still debated. A 2014 meta-analysis by Taylor et al. including nine randomized controlled trails (RCT) and eight observational studies (25) found that gestational supplementation reduced maternal thyroid volume and Tg levels. Half of the RCTs did not show a change in TSH and half demonstrated a mild rise in the control group, thus concluding that supplementation might prevent a rise in TSH. None of the intervention trials recorded an excess frequency of thyroid dysfunction hence demonstrating that iodine supplementation is safe. Another recent RCT from Italy (26) found no difference in maternal TSH during pregnancy, no difference in newborns’ TSH, a slightly lower thyroid volume in the treated group, overall concurring with treatment safety. Interestingly, two observational studies conducted by Moleti et al. found that iodized salt consumption for 2 years prior to pregnancy was associated with lower prevalence of maternal thyroid failure compared to supplementation started at the beginning of pregnancy (27). In contrast, in a study by Abel et al. introducing iodine supplementation after gestational week 12, was associated with lower FT4 and a non-significant lower FT3 suggesting that iodine containing supplements might temporarily inhibit thyroid hormone production and/or release (8). The discrepancies between the different studies can be due to factors such as the timing of performing thyroid function tests during gestation and the timing of introducing iodine containing supplements. Collectively, these data suggest that a population-based iodine fortification plan is a better approach.

The major strength of our study is a well characterized cohort of women with detailed maternal and fetal data. Prospective data collection and questionnaires allowed multiple analyses regarding the factors leading to iodine insufficiency.

The limitations of our study include: 1) The relatively small number of women included and the possibility that a larger cohort would yield different results cannot be excluded. However, thyroid functions in our study were well within the normal range both in mothers and newborns without a noticeable trend; 2) The 24-h recall data might not reflect habitual iodine consumption and we do not have data on iodine containing supplement consumption before pregnancy; 3) We measured thyroid function only once in the first trimester and therefore we cannot exclude the possibility of thyroid dysfunction later in pregnancy; 4) Long-term neurocognitive offspring evaluation is lacking.

In conclusion: Our study adds to a growing body of evidence suggesting that mild-moderate iodine deficiency does not adversely affects maternal thyroid function and has no short-term effects on the newborn. However, more data are needed regarding the possibility of long-term neurodevelopmental effects to guide decision making. Since previous studies support the safety of iodine supplementation, especially when started before pregnancy, in countries with known iodine deficiency and no national fortification plan, the current recommendation to provide iodine supplementation to women seeking pregnancy, still seems to be a safe and reasonable approach.

## Data Availability Statement

The datasets generated for this study are available on request to the corresponding author.

## Ethics Statement

The studies involving human participants were reviewed and approved by the Institutional Review Board (IRB) of Clalit Health Services. The patients/participants provided their written informed consent to participate in this study.

## Author Contributions

TS, TZ, HK, VO designed the study, TS, TZ, VO, HK, GS and AA, data acquisition, analysis, and interpretation. TS, TZ, HK, VO, GS, and AA contributed in the preparing of the manuscript. All authors reviewed the manuscript in detail. All authors contributed to the article and approved the submitted version. All authors agree to be accountable for all aspects of the work in ensuring that question related to the accuracy and integrity of any part of the work are appropriately investigated and resolved.

## Conflict of Interest

The authors declare that the research was conducted in the absence of any commercial or financial relationships that could be construed as a potential conflict of interest.
